# Allelopathic effects of six alfalfa varieties at three stubbles on the germination, seedling and root growth of green foxtail and barnyardgrass

**DOI:** 10.1371/journal.pone.0316137

**Published:** 2024-12-23

**Authors:** Meixuan Li, Xiaohan Gai, Qian Bai, Fanru Xu, Shipu Cheng, Fuhong Miao, Qingwei Liang, Juan Sun, Yufang Xu

**Affiliations:** 1 College of Grassland Science, Qingdao Agricultural University, Qingdao, China; 2 Shandong Key Laboratory for Germplasm Innovation of Saline-alkaline Tolerant Grasses and Trees, Qingdao Agricultural University, Qingdao, China; 3 Key Laboratory of National Forestry and Grassland Administration on Grassland Resources and Ecology in the Yellow River Delta, Qingdao Agricultural University, Qingdao, China; 4 Chifeng Institute of Agriculture and Animal Husbandry Science, Chifeng, China; Shandong University, CHINA

## Abstract

Alfalfa (*Medicago sativa*) is known to release allelopathic substances to affect the germination and growth of other plants, which have the potential to be applied in controlling weeds. Green foxtail (*Setaria viridis*) and barnyardgrass (*Echinochloa crus-galli*), as malignant weeds worldwide, also pose a serious threat to alfalfa in northern China. In this study, the sensitivity of the two weeds to the extracts from the first, second, and third stubbles of six varieties were investigated to further reveal the allelopathic interference of different varieties of alfalfa on notorious weeds. The germination rate, the length and fresh weight of seedlings, the length and fresh weight of roots were measured to elucidate the allelopathy of alfalfa extracts on the two weeds. The results suggested that: (1) The allelopathy of six alfalfa varieties tested showed obvious intraspecific differences, the inhibition of Zhongmu No.3 on green foxtail and barnyardgrass was weaker than other varieties, with the values of synthetical allelopathic effect (SAE) were -0.55 and -0.29, respectively. (2) The inhibitory effect of alfalfa extracts on green foxtail was enhanced with the increase of stubbles, while the differences between three stubbles on barnyardgrass were not clear, especially between the first and second stubbles. (3) Compared with barnyardgrass (SAE = -0.39 ~ -0.29), green foxtail (SAE = -0.65 ~ -0.52) was generally more susceptible to the extracts. (4) The inhibitory effect of alfalfa extracts on root was stronger than seedling in the same weed. For example, the third stubble extracts of Baoding variety inhibited 88.00% of the roots at the concentration of 0.01 g mL^-1^, but did not affect the seedlings of green foxtail. The study may help to comprehensively reveal the allelopathic effect of different alfalfa varieties in the first three stubbles on green foxtail and barnyardgrass, providing scientific evidence for weed control based on natural plant extracts in the future.

## Introduction

Weeds are one of the most important pests in artificially cultivated grasslands [1], which mainly controlled by chemical herbicides. However, the excessive use of herbicides leads to environmental pollution and poses a threat to human safety [2]. Meanwhile, the dependence on chemical herbicides has led to the emergence of herbicide-resistant weed species worldwide [3]. A total of fifteen cases of herbicide-resistant weeds have been reported in alfalfa (*Medicago sativa*) fields globally since 1984, involving ten types of weeds [4], all of which pose greater challenges to the chemical control of weeds in alfalfa fields. Nevertheless, plant-derived allelochemicals were considered as the critical substitutes for traditional chemical herbicides [5,6].

Allelopathy refers to an interference mechanism in which the plant release bioactive metabolites that have direct or indirect beneficial or harmful effects on surrounding plants [7]. Most allelopathic effects are suppressed on surrounding plants and microorganisms by releasing phytotoxic substances [8,9]. Thus, the plant-derived allelochemicals have broad application prospects, and corresponding plant materials with allelopathic effects also urgent to be studied. It was reported that alfalfa has the certain allelopathic effect on itself (autotoxicity) and other plants, and the potential of each variety was not equal. For example, WL656HQ and 3105C were autotoxicity-tolerant and autotoxicity-sensitive varieties in twenty-two alfalfa varieties, respectively [10]. The allelopathic potential in alfalfa plants to lettuce (*Lactuca sativa*) varied with the eight common varieties [11]. Recently, a MYB transcription factor, OsMYB57, has been found to positively regulate rice (*Oryza sativa*) allelopathy and increase its inhibition rate on barnyardgrass (*Echinochloa crus-galli*) [12]. Therefore, the allelopathic mechanism of plants might provide an alternative pathway for weed control that reduces dependence on herbicides, especially for herbicide-resistant weeds.

In recent years, researchers have preliminarily explored the allelopathic effect of alfalfa on some weeds [13–17]. For example, the alfalfa extracts significantly affected seedling growth and root morphology of barnyardgrass [13]. Alfalfa with different genotypes displayed a range of allelopathic interference in annual ryegrass (*Lolium rigidum*) seedlings, and the most phytotoxic alfalfa variety reduced the root length of annual ryegrass by 62% [14]. The germination and seedling growth of various weed species were inhibited by aqueous extracts from dried alfalfa, including lambsquarters (*Chenopodium album*), giant foxtail (*Setaria faberii*), velvetleaf (*Abutilon theophrasti*), pigweed (*Amaranthus retroflexus*), crabgrass (*Digitaria sanguinalis*), cheatgrass (*Bromus secalinus*) and mugwort (*Artemisia vulgaris*) [15,16]. In addition, the alfalfa leaf extracts inhibited the growth of the calluses of crabgrass, lambsquarters, purple amaranth (*Amaranthus lividus*), purslane (*Portulaca oleracea*), and asiatic dayflower (*Commelina communis*) [17]. However, the comprehensive evaluation of varieties, stubbles, and concentrations in alfalfa allelopathy is rare, which is crucial for exploring allelopathic mechanism in alfalfa fields to control weed.

Green foxtail (*Setaria viridis*) and barnyardgrass were malignant weeds in worldwide [18,19], and which seriously restricted the yield and quality of alfalfa in northern China [20,21]. In this study, the allelopathic potential of six excellent alfalfa varieties suitable for planting in northern China to green foxtail and barnyardgrass was evaluated by measuring the 1) germination rate, 2) seedling length, 3) seedling fresh weight, 4) root length, and 5) root fresh weight of weeds under the treatment of the first three stubbles extracts. Based on the above five indicators, synthetical allelopathic effect (SAE) of methanol extracts from the first three stubbles of six varieties on two notorious weeds were also calculated. In summary, this study not only systematically revealed the effects of varieties, stubbles, and concentrations on alfalfa allelopathy, but also provided scientific references for weed allelopathy control.

## Materials and methods

### Preparation of extracts from aboveground parts of alfalfa

The six alfalfa varieties tested were WL319HQ, WL525HQ, WL343HQ, Medoc Max, Baoding and Zhongmu No.3, which were fully proved to be the main varieties suitable for planting in eastern Shandong. All the varieties were naked seed obtained by Beijing Zhengdao Seed Industry Co., Ltd. The seeds of the above different alfalfa varieties were sown in the field of Qingdao (123.16°E, 37.49°N) on March 10^th^, 2018. The parts that exceed 5 cm above the ground was cut on May 17^th^, and the regenerated alfalfa was harvested twice on June 20^th^ and July 15^th^ of the same year. Fresh aboveground parts of alfalfa from first three stubbles were dried to constant weight at 37°C, stored at 4°C after shearing. Accurate 25 g of the above sample was added into 500 mL of 70% methanol solution, and then was oscillated for 12 h at the speed of 150 rpm min^-1^ at 10°C. After 15 minutes of ultrasound, the mixture was repeatedly filtrated by vacuum with 0.45-μm membrane for three times. Subsequently, the collected filtrate was concentrated into the paste by rotary evaporation at 40°C and 50 rpm min^-1^ to eliminate methanol [17,22]. Dissolved the above paste with deionized water and diluted it to 10 mL to obtain an extraction solution with a final concentration of 2.5 g mL^-1^ [17].

### Sterilization of seeds of green foxtail and barnyardgrass

The seeds of green foxtail and barnyardgrass in the research were collected from the alfalfa planting area. The collected weed seeds were washed for three times, and then sterilized with 75% alcohol for one minute. After cleaning with sterile water for three times, immerse the seeds in 30% sodium hypochlorite (NaClO) for 15 minutes, and then clean with sterile water again [23].

### Effects of alfalfa extracts on seed germination, seedling growth, and root growth of green foxtail and barnyardgrass

The treatment solutions with different concentrations (0.01, 0.03, 0.05 and 0.07 g mL^-1^) were prepared by diluting the above extraction solution with sterile water [24,25]. The range of concentration was based on a preliminary study, which showed that alfalfa extracts exhibited a promoting or slight inhibitory effect on weeds at the lowest concentration (0.01 g mL^-1^). A total of 50 sterile weed seeds were evenly placed in a circular petri dish with a diameter of nine centimeters, and then 5 mL of extract solution with corresponding concentration was added. The control group was treated with equal volume of sterile water. The experiments were conducted with three replicates per dose. A total of 540 dishes were used in this study. All the dishes were kept in artificial climate chambers with a regime temperature of 25/20°C (light/dark) and a 16-h photoperiod with a luminous intensity of approximately 15 000 lx. The seed that produced at least 2 mm radicle was considered as germinated [26,27]. The germination rates of seeds were determined five days after germination [28]. At the same time, ten corresponding seedlings were randomly selected from each petri dish, and the length and fresh weight of seedlings and roots were measured respectively.

### Statistical analysis

The response index (RI) of weeds to alfalfa extracts was calculated according to the following formula. In this equal, C and T represent the values of control group and treatment group respectively. If the RI value is positive, the effect of the extracts on weeds is promoted. On the contrary, it is inhibition. The absolute value of RI reflected the magnitude of the allelopathy. The SAE represents the arithmetic mean of the RI of five measurement index of weeds, including germination rate, the length and fresh weight of seedings, the length and fresh weight of roots [29,30]. In addition, the values of SAE on green foxtail and barnyardgrass for each alfalfa varieties with three different stubbles were also calculated respectively.


RI=(T‐C)/T(T≥C)

RI=(T‐C)/C(T<C)

Statistical analysis was performed using SPSS software (v. 20.0, IBM, Armonk, NY, USA), and the data are expressed as mean ± standard deviation (SD). The *Dunnett*’s test at the 5% level of significance in one-way analysis of variance (ANOVA) was carried out to compare the RI values under five concentrations (C0-C4) and three stubbles (S1-S3) in the same variety. Meanwhile, the two-way ANOVA was also conducted on the RI values of each indicator to analyze effects of stubble (S), concentration (C), and their interactions (S × C). In terms of SAE, the differences among the six alfalfa varieties in the same stubble and the differences among the three stubbles in the same variety were compared by one-way ANOVA.

## Results

### Allelopathic effect of alfalfa extracts on the germination of green foxtail and barnyardgrass

**Fig 1:**
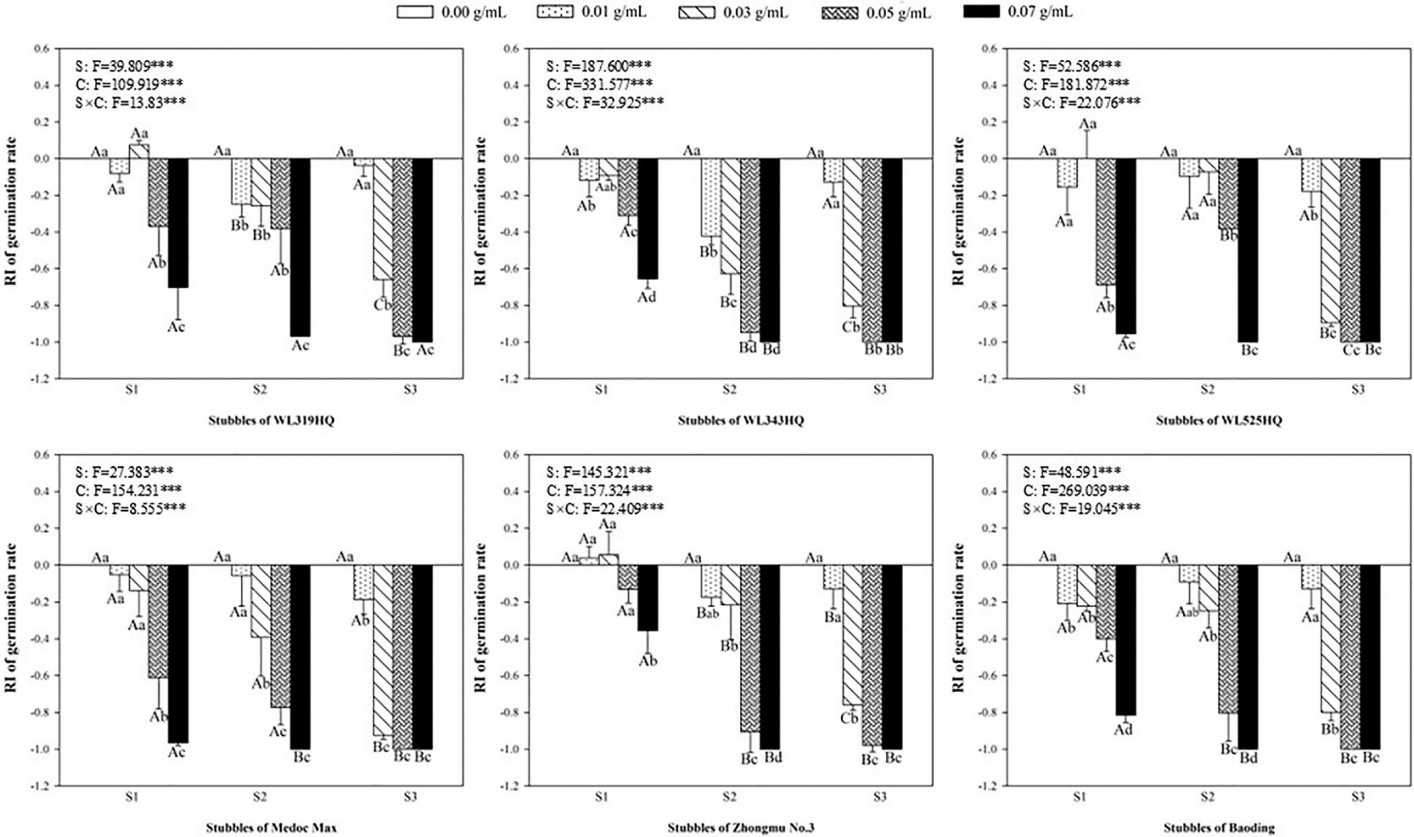
The response index (RI) values of methanol extracts with five concentrations from the first (S1), second (S2) and third (S3) stubbles of six alfalfa varieties to the germination rate of green foxtail. Each data point is the mean ± standard deviation (SD) of three replicates. Different capital (or lowercase) letters in the same variety mean significant differences (*P* < 0.05) among the three stubbles (or five concentrations). The symbol of “*” represents significant differences between the treatments of stubble (S), concentration (C), and their interactions (S × C). Levels of significance are indicated as: “*” means *P* < 0.05, “**” means *P* < 0.01, “***” means *P* < 0.001, the same below.

There was obvious difference in the allelopathy to the germination of green foxtail between different varieties and different stubbles of alfalfa extracts (Fig 1). The germination rate of green foxtail decreased with the increase of the concentration of the extracts. For the extracts of the first stubble of WL319HQ, the germination rate of 0.05 g mL^-1^ and 0.07 g mL^-1^ were 46.67% and 22.00%, respectively, significantly lower than 74.00% of the control (Fig 1). Besides, the concentrations of extracts from different alfalfa varieties that significantly inhibited the germination were also unequal. For the first stubble of alfalfa, when the concentration of extracts from Baoding varieties was 0.01 g mL^-1^, the germination of green foxtail was significantly inhibited, while the extracts from Zhongmu No.3 did not inhibit them until 0.07 g mL^-1^ (Fig 1).

**Fig 2:**
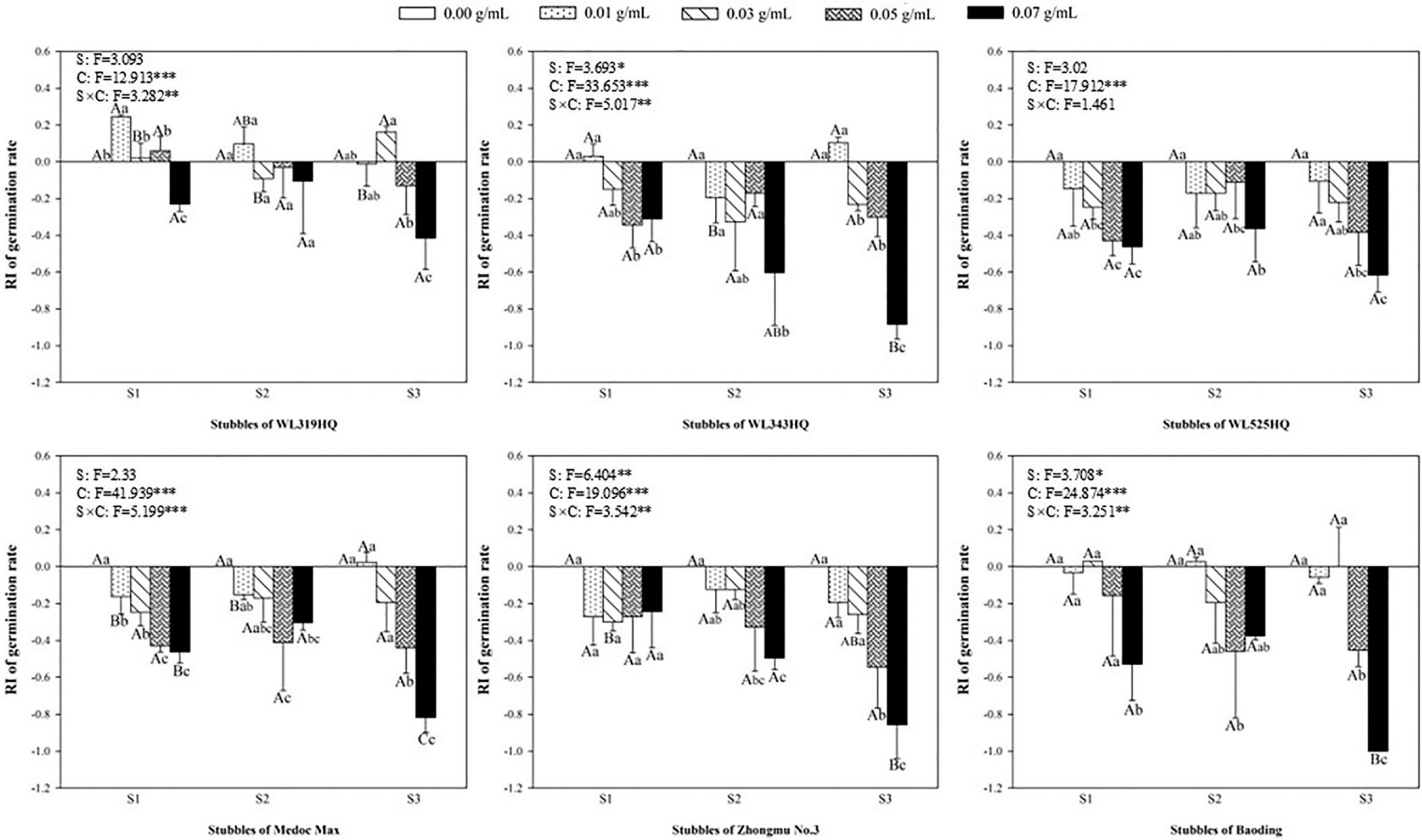
The RI values of methanol extracts with five concentrations from the first (S1), second (S2) and third (S3) stubbles of six alfalfa varieties to the germination rate of barnyardgrass.

Similarly, the allelopathic effects of extracts from different varieties of alfalfa on the germination of barnyardgrass were shown in Fig 2. The low concentration of alfalfa extracts had almost no effect on the germination of barnyardgrass, while only the high concentration inhibited them. Except for Zhongmu No.3, the other five varieties significantly inhibited the germination of barnyardgrass at 0.07 g mL^-1^ in the matter of the first stubble of alfalfa extracts, while WL525HQ and Medoc Max exhibited significant suppression when treated with the lower concentration (0.03 g mL^-1^) (Fig 2). In addition, only the extracts from the first and third stubble of WL319HQ alfalfa significantly inhibited the germination, while the second stubble did not (Fig 2).

### Allelopathic effect of alfalfa extracts on the seedling of green foxtail and barnyardgrass

**Fig 3:**
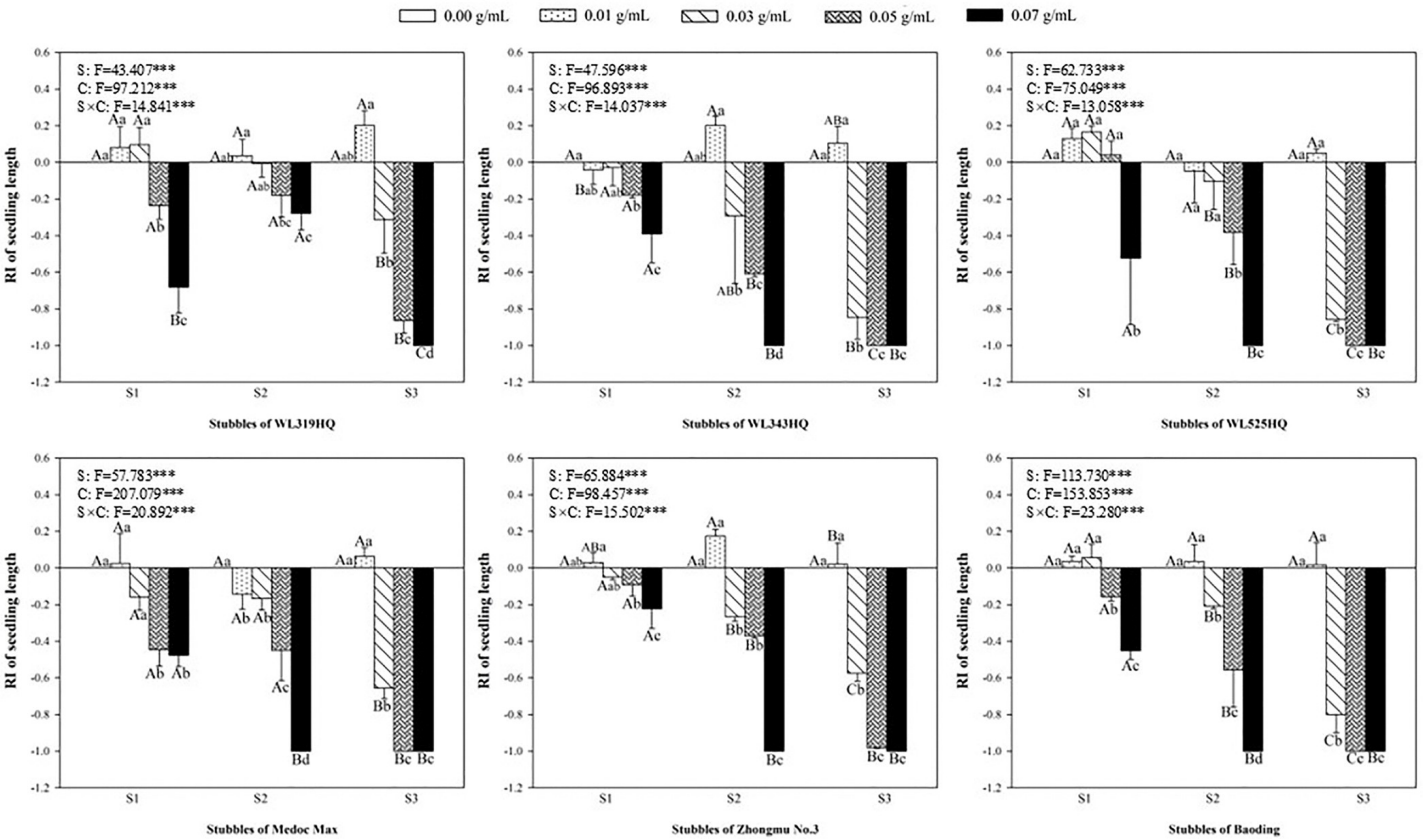
The RI values of methanol extracts with five concentrations from the first (S1), second (S2) and third (S3) stubbles of six alfalfa varieties to the length of green foxtail seedlings.

**Fig 4:**
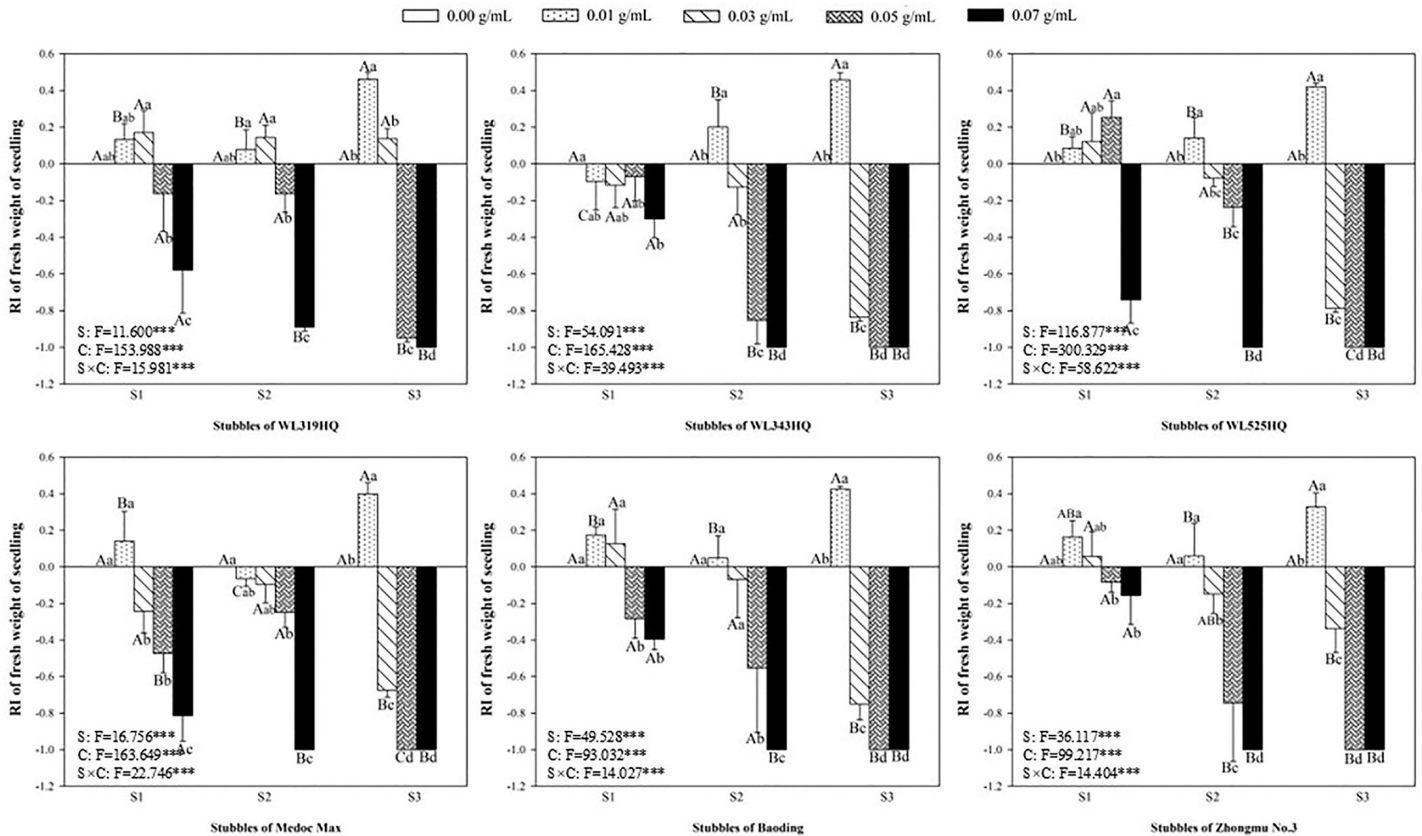
The RI values of methanol extracts with five concentrations from the first (S1), second (S2) and third (S3) stubbles of six alfalfa varieties to the fresh weight of green foxtail seedlings.

**Fig 5:**
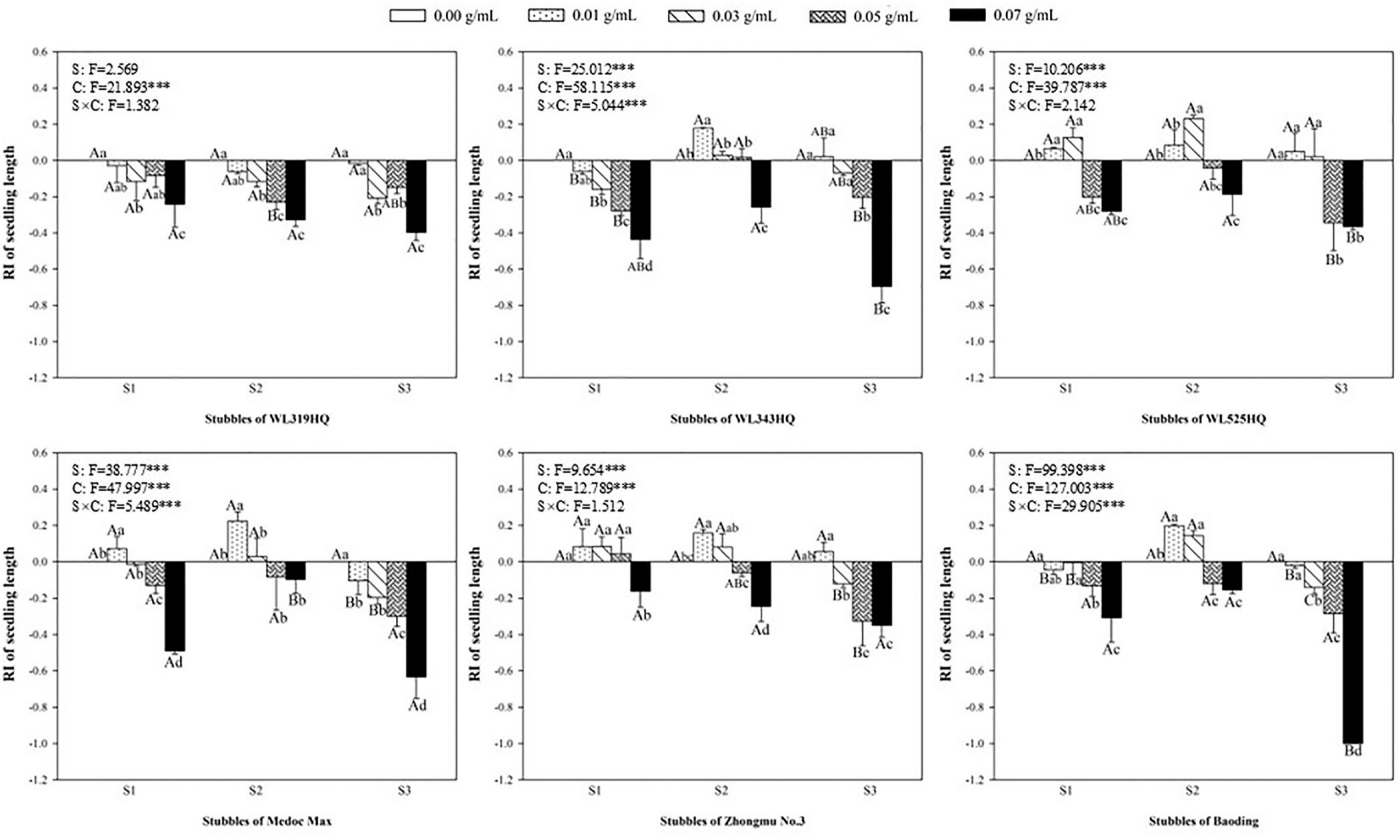
The RI values of methanol extracts with five concentrations from the first (S1), second (S2) and third (S3) stubbles of six alfalfa varieties to the length of barnyardgrass seedlings.

**Fig 6:**
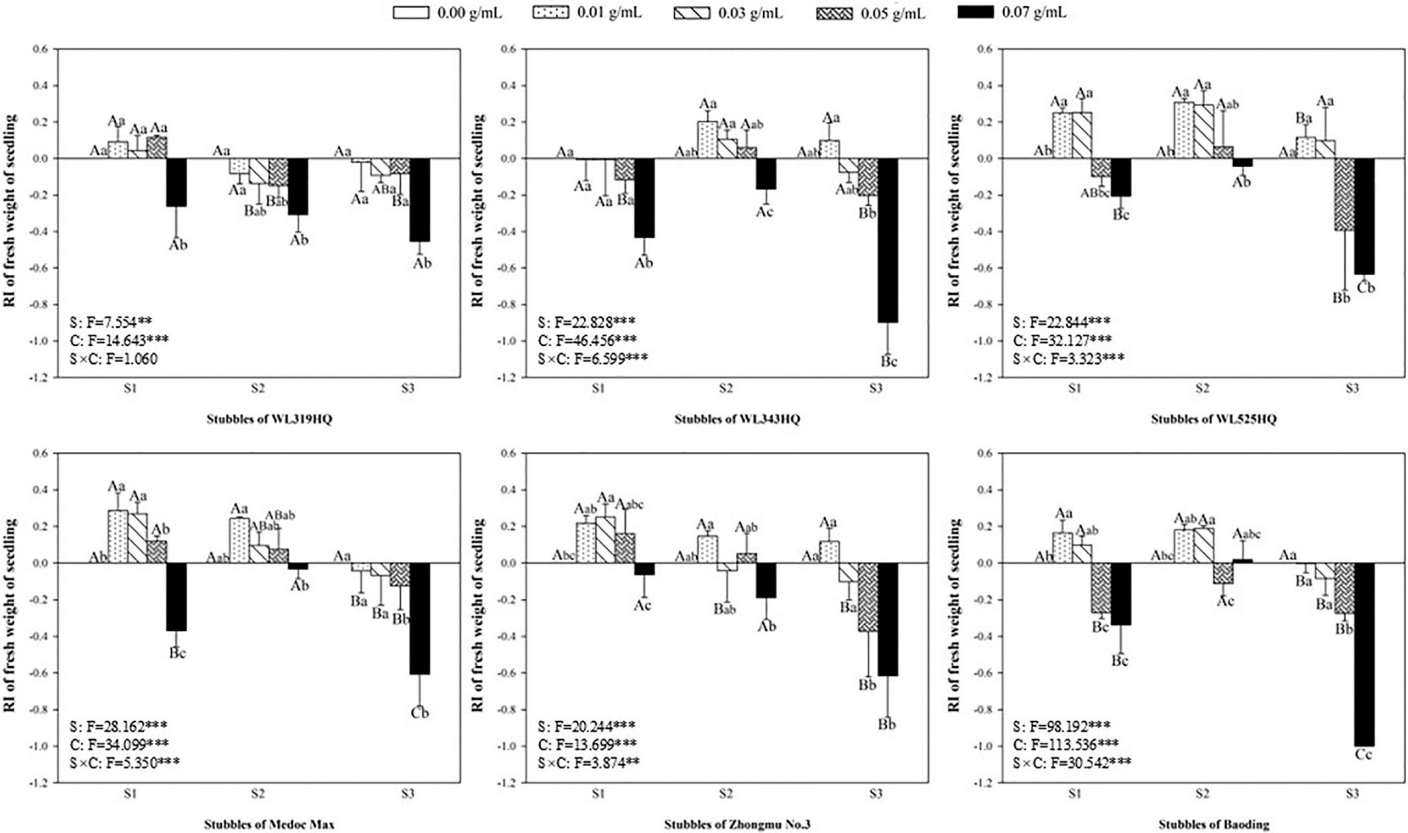
The RI values of methanol extracts with five concentrations from the first (S1), second (S2) and third (S3) stubbles of six alfalfa varieties to the fresh weight of barnyardgrass seedlings.

The length and fresh weight of seedlings were measured to evaluate the allelopathy of alfalfa extracts on the overground part of weeds (Figs 3–6). The inhibitory effects on the seedling of green foxtail increased as the concentration of the alfalfa extracts increased (Figs 3 and 4). The third stubble extracts of all varieties significantly promoted the fresh weight of seedling at the lowest concentration (0.01 g mL^-1^), while the promotion on the length was not significant. However, high concentration of extracts could inhibit the fresh weight or the length of green foxtail. For example, the fresh weight inhibition rate caused by extracts from the third stubble of WL343HQ at the concentration of 0.03 g mL^-1^ was 83.75%. Except WL319HQ, the third stubble extracts of the other five varieties completely inhibited the growth of seedlings at the concentration of 0.05 g mL^-1^, and the second stubble extracts required 0.07 g mL^-1^ (Figs 3 and 4).

The allelopathy of alfalfa extracts on barnyardgrass seedlings was also determined (Figs 5 and 6). Compared with green foxtail, the promotion of low-concentration extracts on the growth of barnyardgrass was not uniform. Only the extracts from the first stubbles of WL525HQ could promote the fresh weight at the lowest concentration. Seedling growth of barnyardgrass was less inhibited by alfalfa extracts than that of green foxtail. The concentration of extracts to inhibit the fresh weight of barnyardgrass was 0.5 or 0.7 g mL^-1^, which was generally higher than that required by green foxtail. Moreover, the extracts of the second stubbles exhibited the weakest inhibitory effect on barnyardgrass, only WL319HQ and WL343HQ obviously inhibited the fresh weight at the highest concentration, and other five varieties did not (Figs 5 and 6).

### Allelopathic effect of alfalfa extracts on the root of green foxtail and barnyardgrass

**Fig 7:**
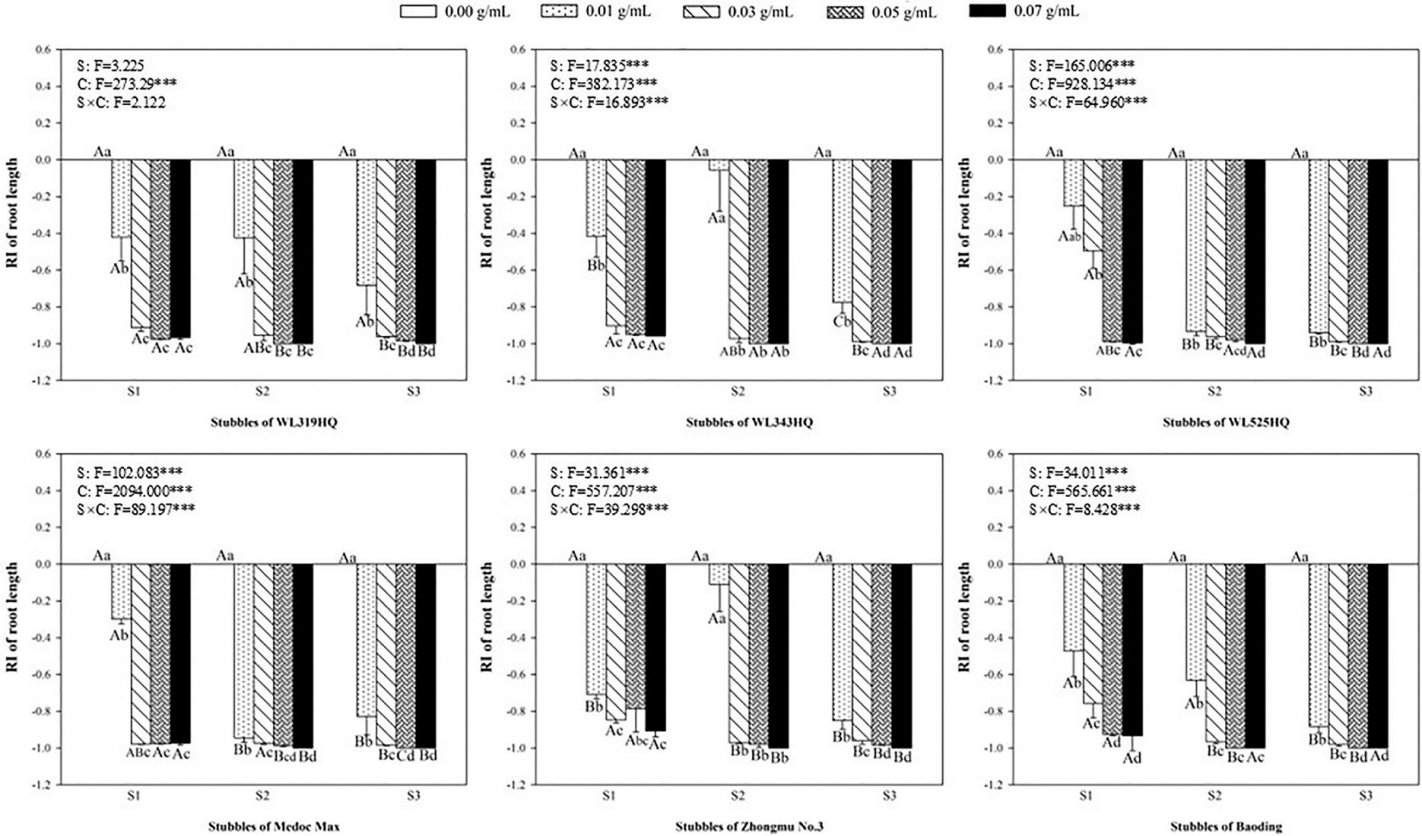
The RI values of methanol extracts with five concentrations from the first (S1), second (S2) and third (S3) stubbles of six alfalfa varieties to the length of green foxtail roots.

**Fig 8:**
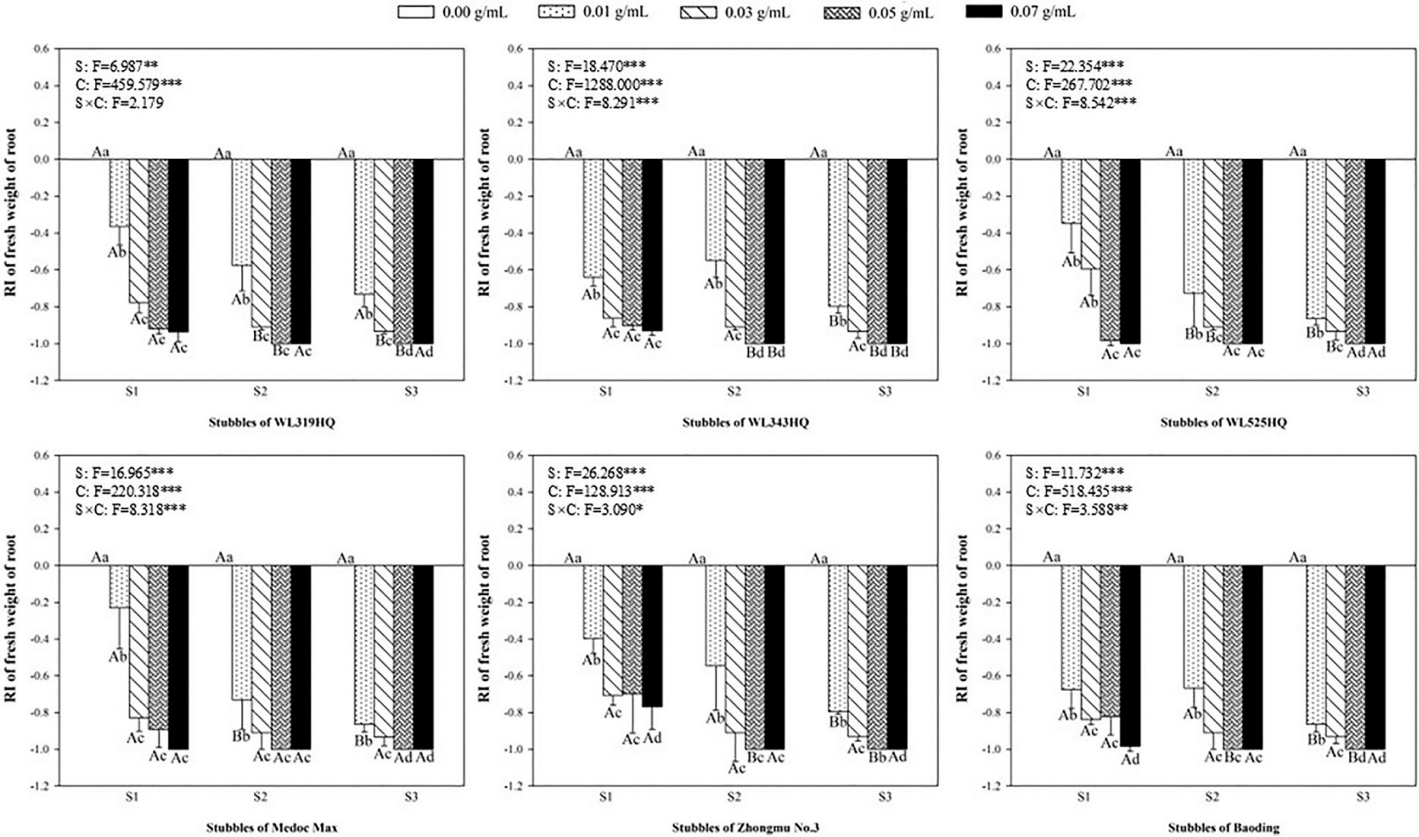
The RI values of methanol extracts with five concentrations from the first (S1), second (S2) and third (S3) stubbles of six alfalfa varieties to the fresh weight of green foxtail roots.

**Fig 9:**
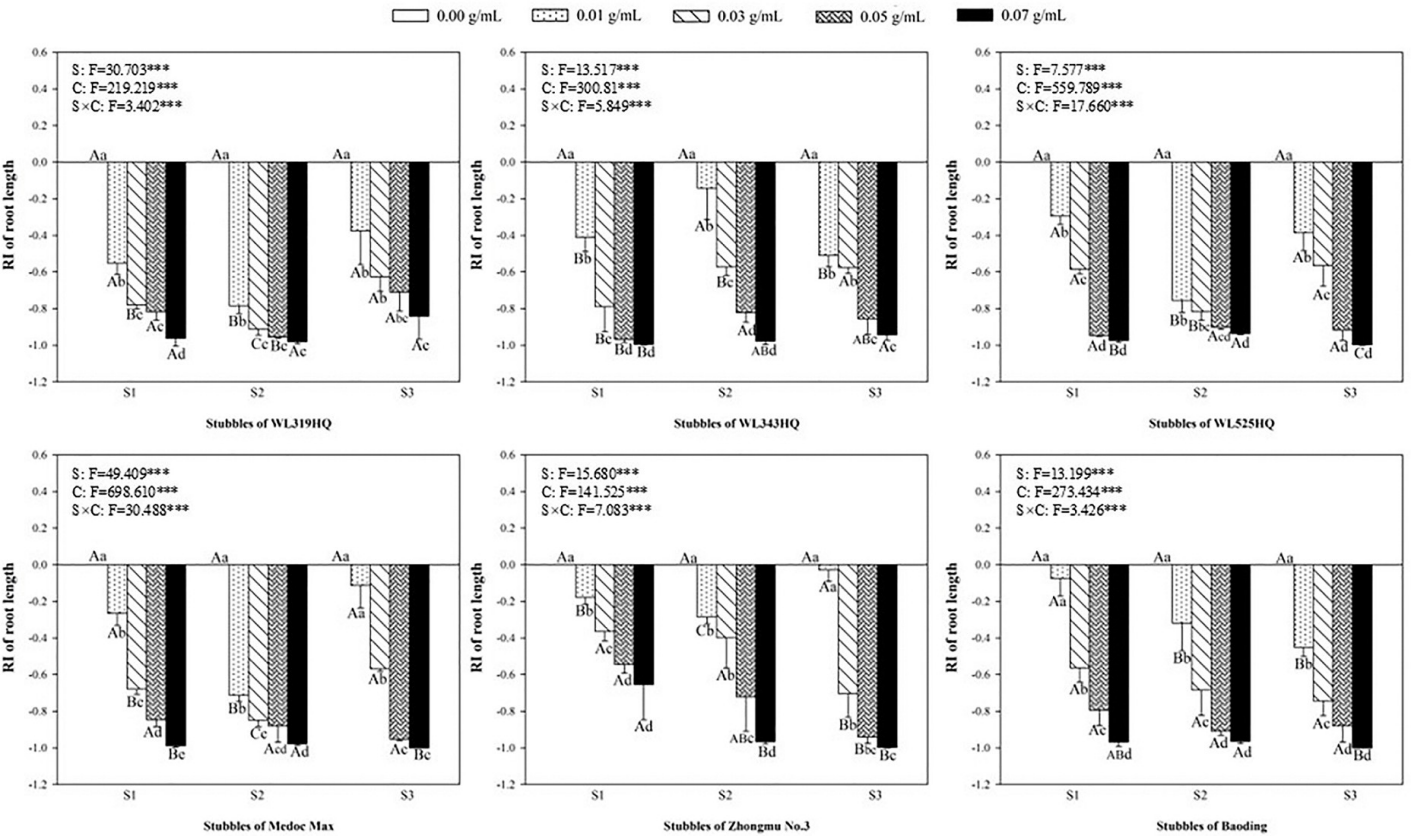
The RI values of methanol extracts with five concentrations from the first (S1), second (S2) and third (S3) stubbles of six alfalfa varieties to the length of barnyardgrass roots.

**Fig 10:**
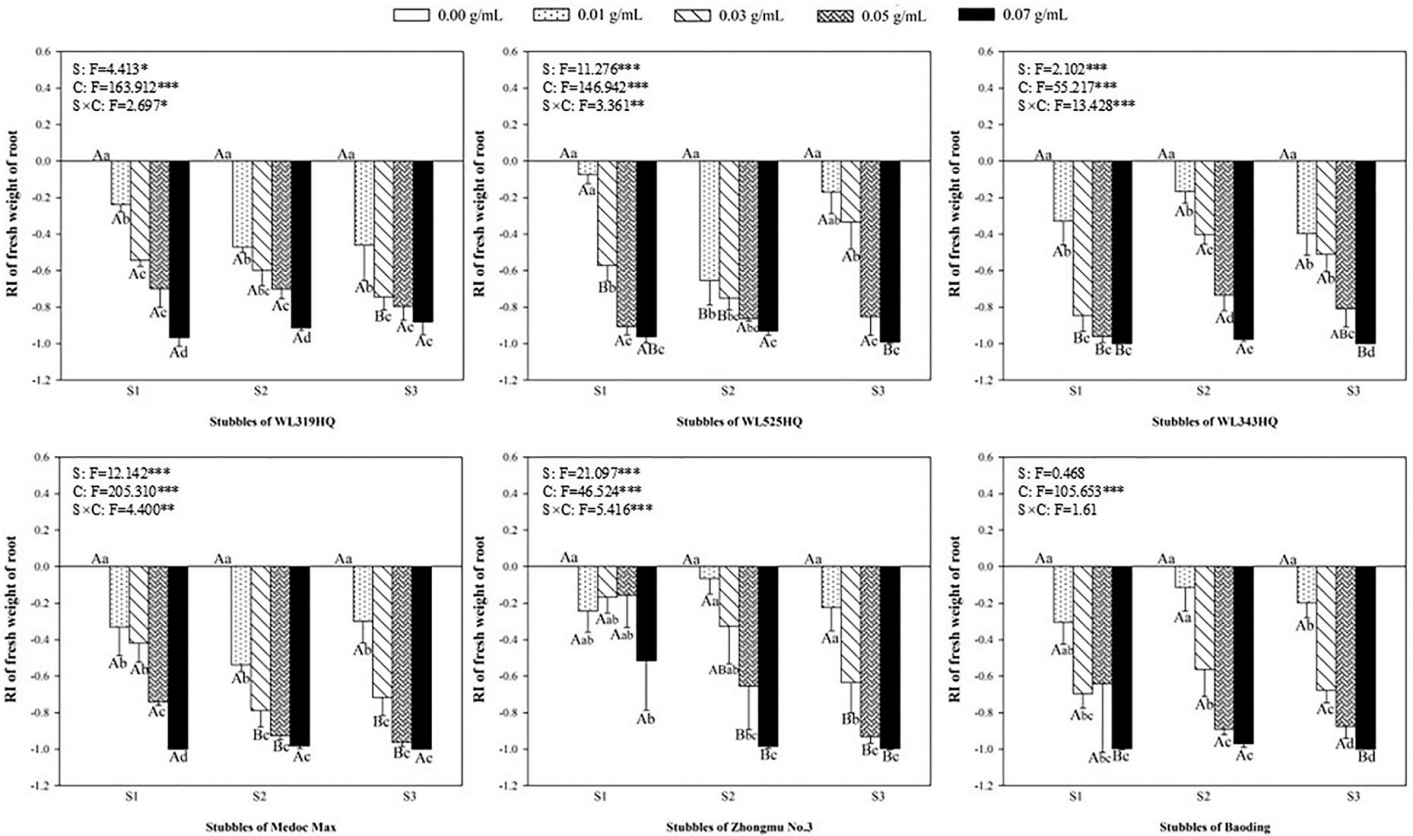
The RI values of methanol extracts with five concentrations from the first (S1), second (S2) and third (S3) stubbles of six alfalfa varieties to the fresh weight of barnyardgrass roots.

Accordingly, the length and fresh weight of roots were used to evaluate the allelopathy of alfalfa extract on the underground part of green foxtail and barnyardgrass (Figs 7–10). The root of the two weeds were more susceptible to the alfalfa extracts than the germination or the growth of seedling. Most of extracts significantly inhibited the length and fresh weight of root of green foxtail at the lowest concentration. The concentration of few extracts that significantly inhibited root growth increased to 0.03 g mL^-1^, such as the second stubble of WL343HQ (Fig 7).

The allelopathic effect of alfalfa extract on the roots of barnyardgrass also was accurately measured (Figs 9 and 10). In general, the allelopathic effects of Zhongmu No.3 and Baoding on the growth of roots were relatively weaker than the other four varieties. As regard to the extract of the first stubble of Zhongmu No.3, the fresh weight of barnyardgrass was not significantly inhibited until the highest concentration. The concentration of the extracts from the second and third stubbles of Zhongmu No.3 that began to inhibit the fresh weight of barnyardgrass was 0.05 g mL^-1^ or 0.03 g mL^-1^, respectively (Fig 10).

### The SAE of the alfalfa extracts on green foxtail and barnyardgrass

**Fig 11:**
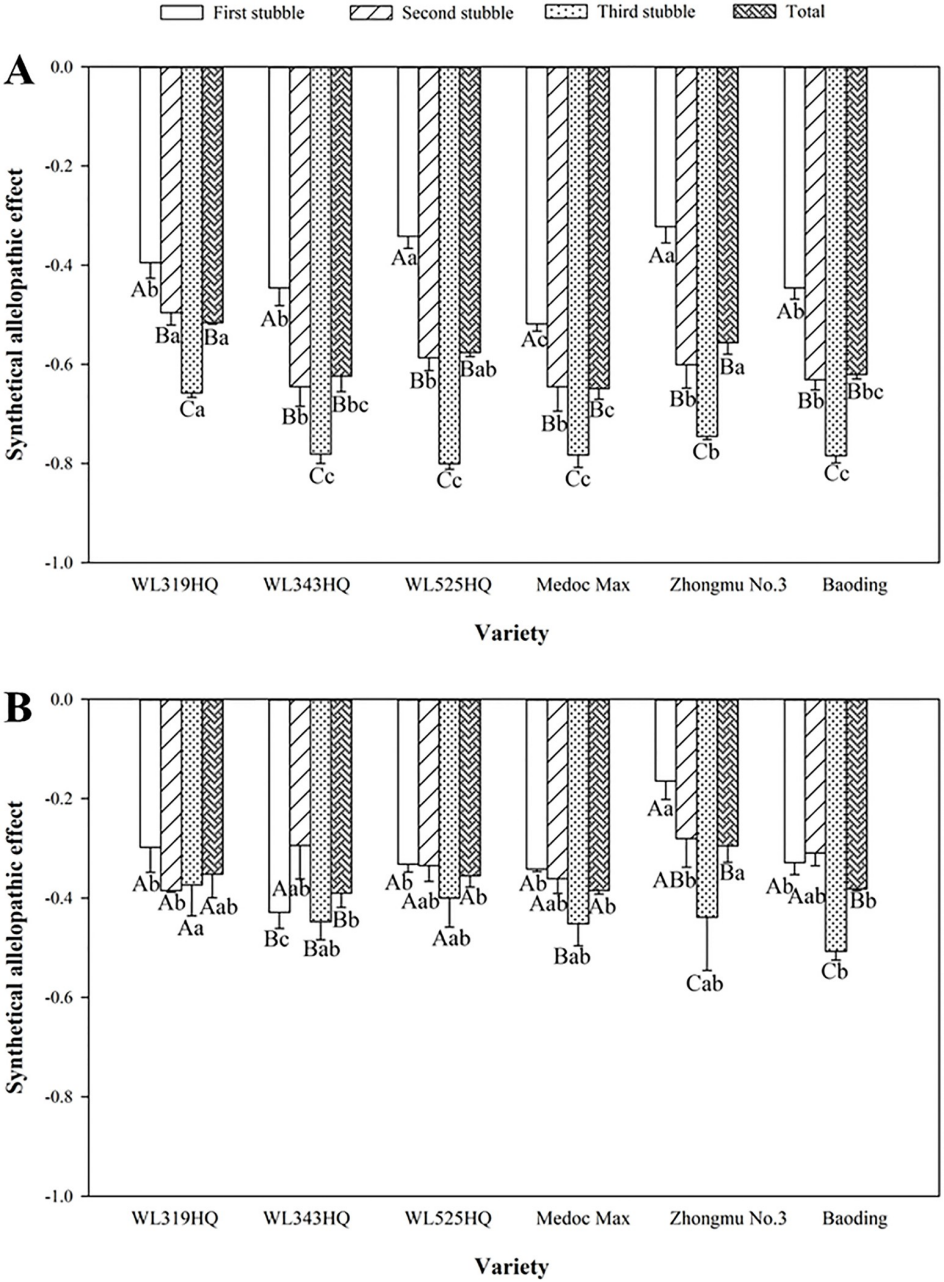
**Synthetical allelopathic effect (SAE) of methanol extracts from the first three stubbles of six alfalfa varieties on green foxtail (A) and barnyardgrass (B).** Different lowercase letters in the same stubble indicate significant differences among the six alfalfa varieties, different capital letters in the same variety indicate significant differences among the three stubbles and their synthesis.

The values of SAE were acquired by all indicators of green foxtail or barnyardgrass treated with extracts from each alfalfa variety (Fig 11). The absolute values of SAE from Medoc Max on green foxtail were generally higher than those of Zhongmu No.3 and WL319HQ. Moreover, the SAE values from three stubbles of the same variety on green foxtail exhibited significant difference, and corresponding absolute values gradually increased with the number of stubbles increased (Fig 11A).

Similarly, the allelopathic effect of the extracts from Zhongmu No.3 on barnyardgrass was also lower than that of other varieties. The SAE value of the first stubble of alfalfa extracts from Zhongmu No.3 was -0.16, which was also far weaker than other varieties. Unlike green foxtail, the differences in the allelopathic effects of barnyardgrass among three stubbles of all varieties was not consistent. For example, there was no difference in the first and second stubbles of five varieties. Even in the three stubbles of WL319HQ and WL525HQ, no significant differences were observed (Fig 11B).

## Discussion

### Intraspecific differences in allelopathic effects of alfalfa

The results in present research showed that the allelopathic effects of alfalfa were significantly different among varieties (Fig 11), together with other published studies in alfalfa [14,28,31]. For example, the Q75 and Titan9 were the least allelopathic genotypes among the 40 alfalfa genotypes when the density was 15 lings/beaker [14]. There were also significant differences in allelopathic effects of aqueous extracts from six different varieties of alfalfa seedling on orchardgrass (*Dactylis glomerata*) [31]. The inhibition effects of Phabulous, Longdong and Zhongmu No.1 on several weeds were stronger than that of other twelve tested varieties [28]. The differences in weed inhibition among different alfalfa varieties provide potential genetic resources for the development of allelopathic alfalfa varieties. The expression of *OsPAL2;3* gene in the allelopathic rice was higher than that in the non-allelopathic rice, and transgenic rice lines with upregulated *OsPAL2;3* expression had higher weed-suppression capacity on barnyardgrass [12]. Compared with allelopathic rice, the allelopathic genes mediating the differences in allelopathy among different alfalfa varieties in this study are not yet clear. If corresponding allelopathic genes and their transcription factors in alfalfa varieties can be combined with high-yield varieties, it will ultimately provide broad prospects for weed control in legume forage fields such as alfalfa.

### Differences in allelopathic effects of alfalfa at different stubbles

There were significant differences in the allelopathic effects of alfalfa on weeds among different stubbles. Compared to the first and second stubbles of Zhongmu No.3, the third stubble showed the stronger inhibition on green foxtail and barnyardgrass, with corresponding SAE values of -0.75 and -0.44, respectively (Fig 11). This is consistent with the reports that the content of allelochemicals accumulated with the increase of planting years of alfalfa [32,33]. On the contrary, Yuan et al (2008) found that younger plants (three-year old) contain more phenolic acids than alfalfa grown in the fourth year [34]. Therefore, the composition of extracts from different varieties at different stubbles maybe complex, and corresponding component analysis will be necessary in the future. Various climate factors, for instance, temperature and precipitation might also play crucial roles in the release and degradation of allelochemicals [35]. The climate changes during the three cutting times may affect the accumulation of allelopathic substances in alfalfa, which may lead to changes in the composition of extracts from different stubbles, not just their content. In addition, more field experiments are needed due to the complexity of the soil environment from the perspective of application.

### Sensitivity differences of different weeds to alfalfa extracts

As is well known, green foxtail and barnyardgrass have been seriously troubling the alfalfa fields in northern China [20,21]. The results pointed to the possibility that the species-specific growth regulatory effects of allelochemicals. Compared with barnyardgrass (SAE = -0.39 ~ -0.29), the corresponding green foxtail (SAE = -0.65 ~ -0.52) in this study was generally more susceptible to alfalfa extracts (Fig 11). The reason for the sensitivity difference between the two weeds was not clear. In fact, the phenomenon of sensitivity difference to alfalfa extracts has also been reported in other weeds. For example, the seedlings of lambsquarters were the most susceptible to alfalfa extracts, and giant foxtail was the most resistant species among six weed species studied [15]. Especially, the two weeds tested in this study all belong to the Poaceae family, and also have significant sensitivity differences. In view of this, although alfalfa has the potential as allelopathic weed control, its application in herbicide spectrum and other aspects still needs further research.

### Sensitivity differences of different parts of the same weed to alfalfa extracts

It was evident that low concentrations of allelochemicals released by alfalfa may stimulate the growth of barnyardgrass or green foxtail. While if the allelochemicals exceed a certain dosage, the growth of weeds will also be inhibited. It was not difficult to find that the growth of root in the same weed was more inhibited than that of shoot (Figs 3–10). The organ specificity of the inhibitory effect also occurs in other weeds such as annual ryegrass and crabgrass [14,25]. The result also corroborated earlier reports that the root growth was more susceptible to extracts than seed germination or hypocotyls growth [13].

In fact, allelochemicals mainly released by underground parts of alfalfa in the field. However, numerous studies have shown that aboveground extracts of alfalfa have a greater inhibitory effect on other plants than underground extracts [15,16]. In order to help identify the key substances or genes in various alfalfa varieties for weed control, aboveground alfalfa was used in this study to evaluate the allelopathic effect on weeds. Meanwhile, more research will be conducted on the allelopathic effects of underground alfalfa on weeds.

## Conclusion

The allelopathic effects of six common alfalfa varieties in northern China on seed germination, seedling growth, root growth of green foxtail and barnyardgrass were comprehensively evaluated in this study. It was found that 1) Medoc Max and Zhongmu No.3 exhibited the strongest and weakest allelopathic effects on the two weeds tested. 2) The inhibitory effect on green foxtail increases with the increase of stubbles for the same alfalfa variety. 3) The stronger inhibitory effect was discovered on green foxtail than that on barnyardgrass. 4) The growth of roots in the same weed was more susceptible to the extracts compared with seedlings. These findings may provide reference for researchers to further explore the mechanisms behind the differences in allelopathic effects of alfalfa in future.
